# Fluorescent Amorphous Distyrylnaphthalene-Based Polymers: Synthesis, Characterization and Thin-Film Nanomolar Sensing of Nitroaromatics in Water

**DOI:** 10.3390/polym10121366

**Published:** 2018-12-10

**Authors:** Raúl O. Garay, Ana B. Schvval, Marcela F. Almassio, Pablo G. Del Rosso, Maria J. Romagnoli, Rosana S. Montani

**Affiliations:** INQUISUR, Departamento de Química, Universidad Nacional del Sur (UNS)-CONICET, Av. Alem 1253, Bahía Blanca B8000CPB, Argentina; abschvval@inquisur-conicet.gob.ar (A.B.S.); almassio@criba.edu.ar (M.F.A.); delrosso@uns.edu.ar (P.G.D.R.); mariajose.romagnoli@uns.edu.ar (M.J.R.); smontani@criba.edu.ar (R.S.M.)

**Keywords:** Segmented conjugated polymer, amorphous, thin films, fluorescent sensor, nitroaromatics, nanomolar detection

## Abstract

Two new fluorescent segmented conjugated polymers with either 1,4- or 2,6-distyrylnaphthalene chromophores and their model compounds were synthesized and the chemosensing abilities of the polymeric thin films to detect nitroaromatics (NACs) in aqueous media were evaluated. The structural, thermal and optical properties of the polymers were correlated with those displayed by their corresponding model compounds. Changes in the connectivity of naphthylene units caused minor differences in optical properties, morphology and quenching efficiencies. Molecular modeling highlighted the extremely bent character of polymer microstructures that explains their high solubility and amorphous character. Polymeric films are amorphous, strongly fluorescent and showed remarkable quenching efficiencies in the nanomolar range with picric acid (PA) and trinitrotoluene (TNT). Quenching experiments using either different nitroaromatic quenchers, excitation wavelengths, excitation beam path-lengths, or time of exposure of the film to the quenching solution evidenced the dominant role of inner filter effects (IFE) in the polymer response to NACs in the micromolar range. The sensing response towards PA, a quencher that strongly absorbs at the excitation wavelength, has an IFE contribution even at the nanomolar range, while the response towards the non-absorbing TNT depends only on the quenching occurring after diffusion of the analyte into the film.

## 1. Introduction

Fluorescent organic materials have a broad range of sensing applications [1,2] and exist in a variety of architectures [3], ranging from small molecules [4] to chromophoric assemblies, such as monolayers [5], macromolecules [6] or dendrimers [7]. Among them, thin films of fluorescent polymers have raised much interest for chemo-/bio-sensing [8] due to their ease of fabrication, mechanical and chemical stability, tunable shape and portability, as well as their success in detecting vapors of explosive compounds as a result of an amplified response [9]. In addition to the intrinsic electro-optical properties of the polymer, permeation of analytes across polymer films, that depends on both morphology and polymer-analyte interactions, is essential in solid-state sensing configurations where luminescent films respond to trace amounts of analytes which are either in the vapor phase or in solution [8,9].

We have previously evaluated the capabilities of thin films of segmented conjugated polymers (SCPs) to detect environmental pollutants presenting a hazard to ecological and human health that are present in water bodies of industrial and city areas, such as nitroaromatic compounds (NACs) [10,11]. In order to obtain amorphous morphologies that could favor diffusion of the analyte from the aqueous solution into the polymeric film, we knitted semi-rigid fluorophores with isopropylene units, which confer a highly twisted nature to the main chain. We found that thin films of these disordered assemblies of fluorophores proved to be very sensitive towards NACs in aqueous media [12,13]. Indeed, we and many others have found that different fluorescent materials [14,15,16,17,18,19,20,21,22,23,24], despite their huge disparity in chemical structure, are very responsive in aqueous media towards picric acid (PA) and show no, or a small, responses to trinitrotoluene (TNT) and related nitrotoluenes. Nevertheless, it has been difficult to establish general structure-property relationships for the quenching responses of NACs that could be used to improve the performance and selectivity of the materials by rational design. Some of the difficulties arise from the occurrence of different or competing quenching mechanisms other than photoinduced electron transfer (ET) [3], such as resonance energy transfer (RET) [3,16] or inner filter effects (IFE) [25,26,27].

**Scheme 1:**
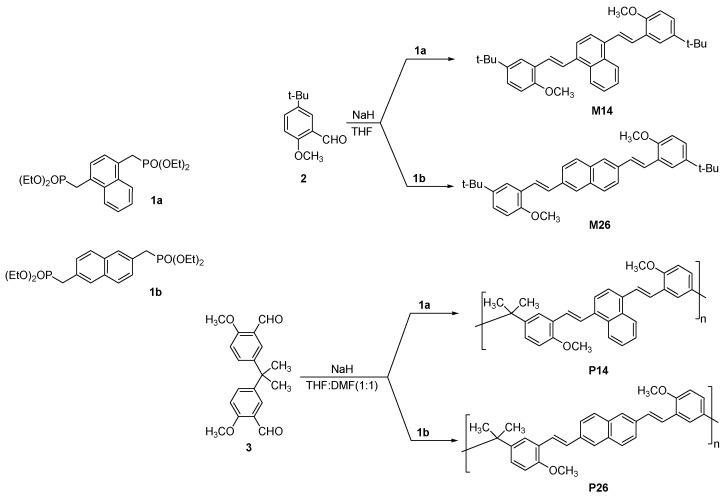
Synthetic routes to polymers and their model compounds.

In this report, we first describe the synthesis and characterization of the fluorescent segmented conjugated polymers **P14** and **P26**, with either 1,4- or 2,6-distyrylnaphthalene chromophores, and their model compounds **M14** and **M26** (Scheme 1). In addition to showing remarkable photophysical properties, the distyrylnaphthalene moieties can be tethered onto the main chain with different regio-connectivity, which could modify optical properties, morphology and quenching efficiencies. The new polymers and their model compounds were characterized by gel permeation chromatography (GPC), differential scanning calorimetry (DSC), NMR spectroscopy, FT-IR spectroscopy, UV-Vis spectroscopy, and fluorescence spectroscopy. The structural, thermal and optical properties of the polymers were correlated with those displayed by their corresponding model compounds. Finally, we evaluated the chemosensing abilities of the polymeric thin films to detect various hydrophilic and hydrophobic nitroaromatics in aqueous media.

## 2. Materials and Methods

### 2.1. General Methods and Instrumentation

The general methods and instrumentation used in the structural, thermal and optical characterization are reported in Supplementary Materials. Several hydrophobic (i.e., 2,4,6-trinitrotoluene, TNT; 2,4-dinitrotoluene, DNT; 4-nitrotoluene, NT) and hydrophilic (i.e., picric acid, PA; 2,4-dinitrophenol, DNP; 4-nitrophenol, NP) nitroaromatics were used. Fluorescence control experiments were previously done by checking during 24 h the occurrence of polymer leakage from the film towards the aqueous media; **P14** and **P26** films supported on quartz or glass plates showed no bleeding.

### 2.2. Materials and Synthesis

Reagents (Sigma-Aldrich, Darmstadt, Germany) were used without further purification. DMF was dried over molecular sieves and stored under argon atmosphere. THF was distilled from Na/benzophenone. 1,4-Bis(bromomethyl)naphthalene and 2,6-Bis(bromomethyl)naphthalene were obtained by radical bromination of 1,4-dimethylnaphthalene and 2,6-dimethylnaphthalene, respectively. The reaction of the dibromides with triethylphosphite afforded the bisphosphonates **1a** and **1b** [28]. Aldehyde **2** and dialdehyde **3** were prepared according to the literature [13]. The details of the synthesis and characterization of the synthetic precursors, monomer, model compounds **M14** and **M26** and the polymers **P14** and **P26** are presented in Supplementary Materials.

### 2.3. Molecular Modeling

The ORCA 4.0 program package (Max Planck Institute for Coal Research, Mülheim, Germany) [29] and the graphical interface Gabedit 2.4.8 (Université Claude Bernard Lyon1, Villeurbanne, France) [30] were used. Initial structures were obtained with the AM1 method and then structure optimization was carried out using the PBEh-3c method [31]. This DFT method is based in a modified PBE functional, uses Ahlrichs-DZ basis sets and the “3c” stands for the use of a gCP correction, a dispersion correction (D3) and minor modifications of the basis sets. It has been designed to compute structures and interaction energies of large systems and could reach results close to that of the MP2/TZ level with a small fraction of the computational cost.

## 3. Results and Discussion

### 3.1. Synthesis

Synthetic routes for the new polymers **P14** and **P26** with bis(styrylnaphthylene) units tethered by isopropylene units and its model compounds **M14** and **M26** are shown in Scheme 1. The Horner-Emmons-Wittig coupling to obtain carbon-carbon double bonds with predominant *E* configuration was used. First, bisphosphonates **1a** and **1b** were prepared from the 1,4- and 2,6-bis(bromomethylene)naphthalenes, respectively. Then, monoaldehyde **2** and dialdehyde **3** were used as coupling partners of **1a** and **1b** to obtain **M14** and **M26** and the polymers **P14** and **P26**. No precipitate or gel formation was detected when THF:DMF (1:1) was used as the polymerization reaction media. But early precipitation and lower degrees of polymerization were observed when the coupling reaction was run in pure THF. Benzaldehyde and diethyl benzylphosphonate were used as end-capping compounds to terminate polymerizations. Despite the lack of solubilizing long alkyl side chains, **P14** and **P26** were soluble in organic solvents such as CHCl_3_, CH_2_Cl_2_, DMF, toluene or THF so the crude polymers were precipitated from their chloroform solutions with methanol to give yellow solids.

**Figure 1:**
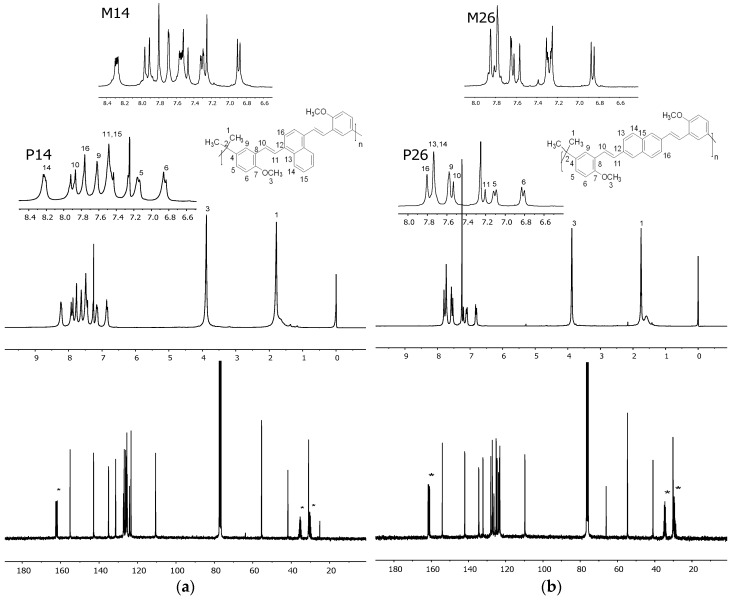
^1^H NMR (CDCl_3_) (top) and ^13^C NMR (CDCl_3_ with 5% DMF) (bottom) spectra of polymers (**a**) **P14** and (**b**) **P26**. The ^1^H NMR spectra of the expanded aromatic region of both model compounds and polymers are also shown. * ^13^C signal from DMF-*d*_7_.

GPC analysis indicated that good degrees of polymerization were reached (**P14**, *M_n_* = 18.7 kDa, *M_n_*/*M_w_* = 2.4, *DP_n_* = 43; **P26**, *M_n_* = 5.5 kDa, *M_n_*/*M_w_* = 2.4, *DP_n_* = 13). NMR and FTIR spectroscopies confirmed the all-trans stereochemistry and chemical structures of the coupling products (Figure 1). The ^1^H spectra of **P14** and **P26** showed ethenyl CH signals above 7.00 ppm with coupling constants *J_trans_* = 16.1 Hz in **P14** and *J_trans_* = 13.5 Hz in **P26**, while no signals were observed in the region of 6.5 ppm which would correspond to their cis-counterparts (Figure 1). Likewise, when compared to their respective polymeric counterparts, the expanded aromatic regions of the model compounds **M14** and **M26** showed quite similar values of chemical shifts and *J*_trans_ coupling constants and no cis-ethylenic signals at ca. 6.5 ppm, thus confirming the structural regularity of the polymeric structures (Figure 1, expanded aromatic regions). Besides, the presence in all coupling products of an intense IR band at ≈960 cm^−1^ (out-of-plane C–H deformation band) of trans-substituted vinylene groups and the absence of the characteristic absorbance of cis-substituted alkene at ≈730 cm^−1^ were additional indications of the high content of trans-configurations.

### 3.2. Morphology. Thermal and Optical Properties

**Figure 2:**
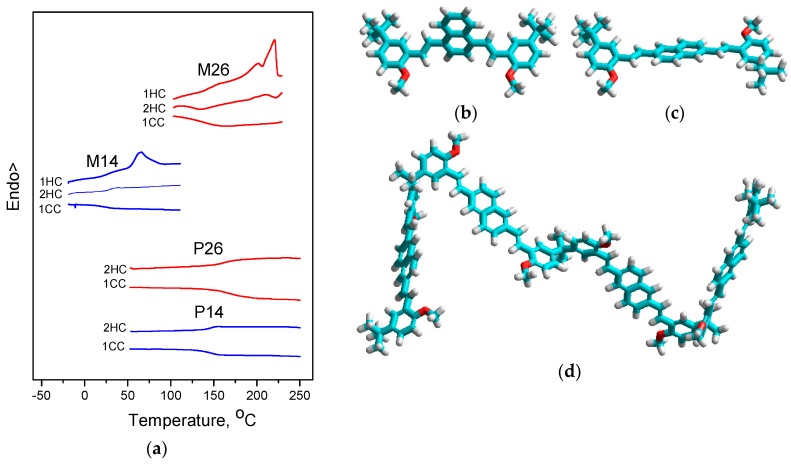
(**a**) Differential scanning calorimetry (DSC) traces of model compounds and polymers. (**b**) DFT/PBEh-3c relaxed geometry of **M14**, (**c**) **M26** and (**d**) a tetramer of **P26**.

The results of the DSC analysis are shown in Figure 2a and Table S1 (Supplementary Materials). The new distyrylnaphthylene **M14** exhibited a strong glass transition (29 °C) and a broad melting endotherm (74 °C) in the first heating cycle, but cooling of the melt led to the formation of a glassy state (19 °C) which persisted in the subsequent heating cycles. **M26** showed a strong glass transition (140 °C) and a broad melting endotherm (220 °C) in the first heating cycle. A broad crystallization exotherm (167 °C) was present only in the first cooling which was followed by the glass transition. Then, **M26** exhibited only strong glass transitions upon repetitive heating and cooling cycles in DSC plots. Occasionally, weak cold crystallization followed by weak melting transitions was observed in the heating cycles. Both compounds bearing bulky tert-butyl groups have strong tendencies to form disordered gatherings that render kinetically stable amorphous glasses, though their differences in molecular structure are manifested in that **M14** forms amorphous glasses after the first heating cycle while **M26** achieves the amorphous state after the second heating cycle. On the whole, the longer and more planar **M26** (Figure 2b) displayed higher transition temperatures than the shorter and bulgy **M14** (Figure 2c). DSC traces of polymers **P14** and **P26** showed only noticeable glass transitions at 155 and 170 °C respectively, these transition values reflect the difference in connectivity of naphthylene groups in the semi-rigid moiety of the polymers again. Moreover, the films appeared completely black when observed by POM with cross polarizers in the temperature range between 50 and 300 °C thus indicating the occurrence of optically isotropic phases (Figure S1, Supplementary Materials). Thin films (thickness range = 30 to 200 nm) cast from chloroform solutions were transparent, homogeneous and appropriate for optical measurements. Likewise, thicker films continued to be transparent without a sign of birefringence even after several weeks at room temperature. Evidently, the high solubility and amorphous nature of the polymers **P14** and **P26** are rooted in the twisted chain conformations that the polymer microstructure must adopt by virtue of the angle imposed to the gem-chromophores by the saturated isopropylene spacer. The DFT/PBEh-3c molecular model of a tetramer of **P26** shown in Figure 2d highlights its highly bent character.

**Figure 3:**
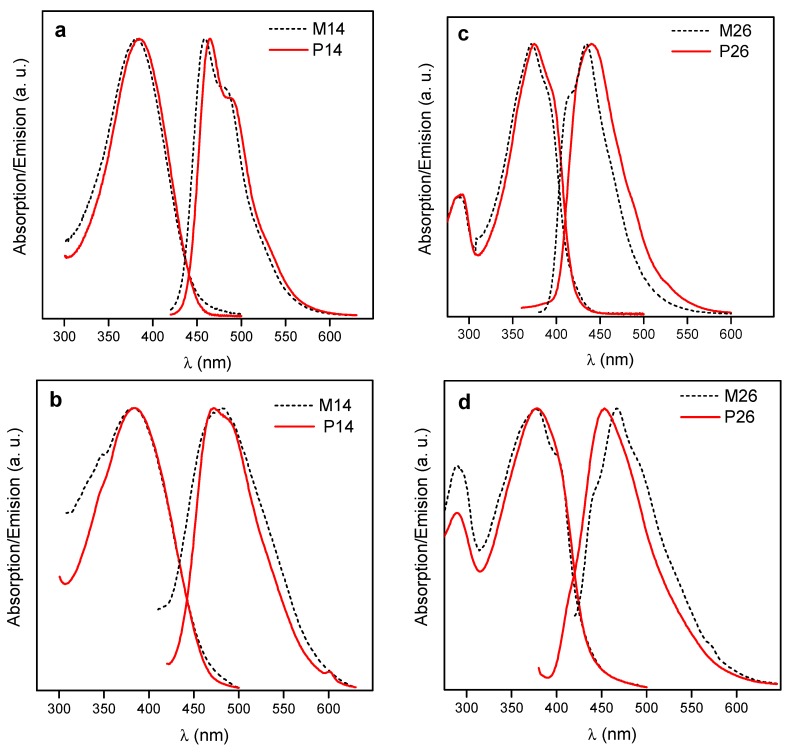
Absorption (left) and emission (right) spectra. (**a**,**c**) CHCl_3_ solutions, (**b**,**d**) Thin films.

The morphological information from DSC and POM was complemented with UV-Vis and fluorescence spectroscopy data that provided information about the interaction and local ordering of the chromophores [32,33]. Absorption and emission spectra of **P14** in chloroform exhibited small red shifts in comparison to those of **M14** (Figure 3a and Table S1, Supplementary Materials). Therefore, the interaction of adjacent chromophores along the isolated polymer chains is quite small. Both absorption spectra, which are usually very informative about the degree of order-disorder present in the solid state, of **M14** (λ_max,abs_ = 383 nm) and **P14** (λ_max,abs_ = 382 nm) films overlap in the condensed phase and show no distinctive differences against those recorded in solution (Figure 3b). Hence, no significant amounts of chromophores in either the monomeric or the polymeric assemblies form local ground-state aggregates within films. Moreover, the fluorescence spectra of a **P14** (λ_max,em_ = 472 nm) film exhibits a small blue-shift against that of **M26** (λ_max,em_ = 482 nm), which is due to a shift in the relative weight of the vibronic bands, revealing that excited species generated by chromophores locked in random orientations by the twisted nature of the polymer chains exist in an environment less ordered than that generated by the monomeric chromophores [32,33]. The same conclusions were reached by a similar comparative analysis of the optical properties of chloroform solutions and films of **M26** and **P26** (Figure 3c,d). Remarkably, all absorption transitions, as well as the emission transitions, of these distyrylnaphthalene-based model compounds and polymers lie roughly in the same spectroscopic region in the thin films showing that the different connectivity did not bring profound changes to their optical behavior. Calculations at the DFT level also indicate that minimal differences in the frontier molecular orbital (FMO) energies of the fluorophores are brought by either the different connectivity of the aromatic moieties in **P14** and **P26** or the monomeric versus the oligomeric structure (Figure 5a).

### 3.3. Fluorescence Response to Nitroaromatic Compounds

**Figure 4:**
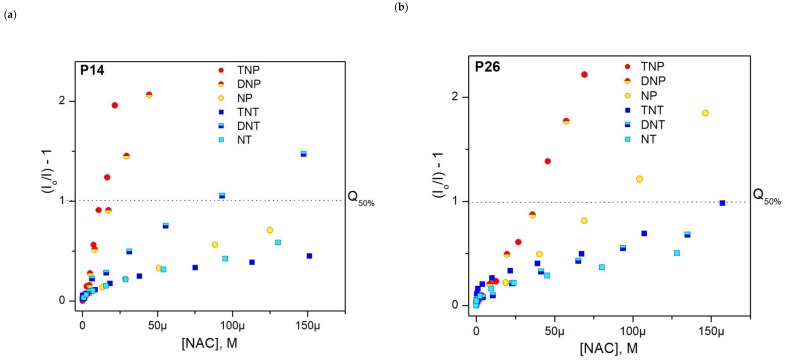
The Stern–Volmer plots with nitrophenols and nitrotoluenes (**a**) for **P14** films (λ_exc_ = 384 nm, λ_obs_ = 470 nm) and (**b**) for **P26** films (λ_exc_ = 371 nm, λ_obs_ = 461 nm). The graphs include a visual aid to indicate the half of the maximum quench, Q_50%_.

Quenching experiments were performed on polymer thin films supported on quartz or glass substrates which were immersed in water, and their fluorescence response to increasing concentrations of NACs was recorded. Data presented in Figure 4 were obtained from fluorescence intensities recorded after signal stabilization in the conditions commonly used to gather fluorescence data, that is, films were irradiated (λ_exc_) at or near the maximum of the lowest energy absorption band (λ_max,abs_) and fluorescence intensities were observed (λ_obs_) at or near the wavelength corresponding to the maximum in the emission spectra (λ_max,em_) of the sensing polymer. The Stern–Volmer (S–V) relationship, (*I*_o_/*I*) − 1 = *K*_SV_ [NAC], between the ratio of the fluorescence intensity with and without the NAC, I and I_o_, and the added analyte concentration, [NAC], was used to quantify the quenching efficiencies for various NACs. Thus, Figure 4 shows plots of the S–V equation for **P14** and **P26** with nitrophenols and nitrotoluenes in the 0–450 µM concentration range. Table S2 (Supplementary Materials) lists the S–V constant (K_SV_) obtained from the linear part of the curves presented in Figure 4, which corresponded to regions between 5%–30% to 55%–65% of the total quenching, and the values of half of the maximum quench, Q_50%_, that is, the quencher concentration needed to reach (*I*_o_/*I*) − 1 = 1.

Polymers **P14** and **P26** showed high sensitivity to increased concentrations of NACs. Despite their microstructural differences, but in line with the similarity of the optical and morphological properties of their films, both polymers displayed fairly comparable responses. The quenching response to nitrophenols is distinctively higher than that to nitrotoluenes for both polymers, with PA and DNP showing the highest sensitivities. Thus, we found that the quenching efficiency order for the most studied NACs is PA >> TNT, as has been frequently observed in many other systems [14,15,16,17,18,19,20,21,22,23,24]. We also noticed that non-linear S–V relationships with upward curvature occurred with nitrophenols at the higher end of quencher concentrations. While with most nitrotoluenes, a rather steep increase of the efficiency in the low micromolar region is followed by a downward curvature at higher concentrations, this phenomena is more noticeable in **P26** (Figure S2, Supplementary Materials). Moreover, in this region quenching efficiencies were comparable, i.e., TNT ≥ PA.

**Figure 5:**
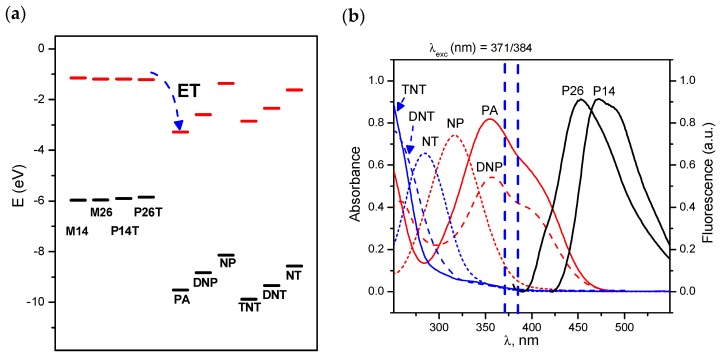
(**a**) Frontier molecular orbitals energies (DFT/PBEh-3c) of **M14**, **M26** and tetramers (**P14T** and **P26T**) of **P14** and **P26**. Values for the frontier molecular orbitals (FMOs) of the quenchers are also included. (**b**) UV-Vis absorption spectra, [NAC] = 56 mM. Fluorescence spectra of **P14** and **P26** are also included. Vertical dashed lines indicate the excitation wavelengths used to record fluorescence spectra.

The non-linearity of S–V plots and the variation in quenching efficiencies between the low micromolar and high micromolar range imply that concurrent quenching mechanisms are operative. In electron-rich structures such as **P14** and **P26**, fluorescence quenching by nitroaromatics that follows their optical excitation is generally attributed to electron-transfer quenching (ET) occurring from the excited polymer to the LUMO of the electron deficient nitroaromatic molecule. In our case, the results of the DFT calculations (see Figure 5a) indicate that ET from the excited polymer to the ground state of the nitroaromatics is thermodynamically favorable for all quenchers. Another mechanism of non-radiative excited state depletion involves radiationless energy transfer processes (FRET) [3,34]. Thus, fluorescence quenching could result from either ET or FRET or a combination of both processes. The latter process requires spectral superposition of the absorption spectrum of the quenchers and the fluorescence spectra of the fluorophores. Figure 5b presents the absorption spectra of the nitrophenols and the nitrotoluenes obtained at the same quencher concentration and the fluorescence spectra of both **P14** and **P26**. There it can be observed that among the NACs tested here only the strongly acidic DNP and PA present intense absorption bands with λ_max,abs_ ≈ 355 nm, which partially overlap the fluorescence spectra of **P14** and **P26**, indicating that FRET could be contributing to quenching with these two NACs. On the contrary, no such overlap occurs with NP and the other nitrotoluenes tested here. Moreover, inner filter effects (IFE) must also be considered for absorbing quenchers. Although IFE is not strictly a fluorescence quenching process, a non-fluorescent quencher could absorb either the excitation beam (primary IFE) or the emitted beam (secondary IFE) of the fluorophores or both. Such attenuation of the excitation and emission beams results in an additional contribution to the total response of the polymers to NACs [34]. The quenching efficiency order for **P26** (see Figure 4 and Table S1, *Q*_50%_ = PA > DNP > NP >> NT > DNT > TNT) follows the intensity of their optical densities (OD) at the excitation wavelength of **P26** (λ_exc_ = 371 nm) as highlighted by the dashed vertical bar in Figure 5b. It also can be appreciated in Figure 5b that the red shift in the λ_exc_ used for **P14** (λ_exc_ = 384 nm) reduces the OD of the quencher NP at the λ_exc_ significantly, and its quenching efficiency also drops accordingly.

**Figure 6:**
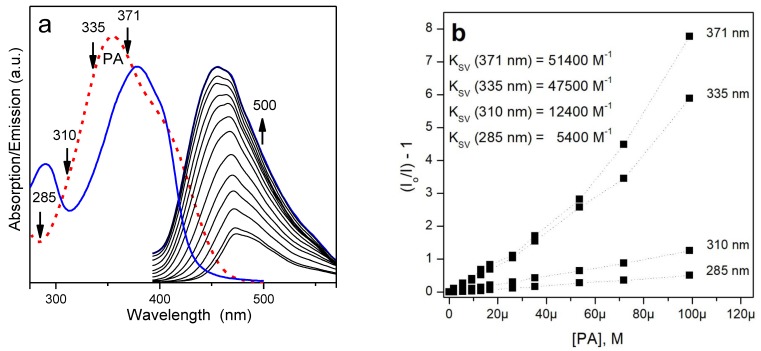
(**a**) Absorption spectrum of the picric acid in water (red dashed line). Absorption spectrum of a **P26** film (solid line) and fluorescence spectra change (λ_exc_ = 371 nm) as a function of increasing concentrations of picric acid (PA) in water, fluorescence spectra were recorded 1 min after the quencher addition. Down arrows indicate the λ_exc_ used to gather data presented in (**b**) while the up arrow indicates the λ_em_ used to observe the fluorescence decrease. (**b**) The Stern–Volmer plots for a **P26** film (λ_em_ = 500 nm) and PA obtained with λ_exc_ = 285, 310, 335, and 371 nm.

Thus, the role of the excitation wavelength in the quenching efficiencies was examined in more detail. Figure 6b shows the S–V plots of the fluorescence response of **P26** to PA, which were obtained with four different excitation wavelengths, λ_exc_, as shown in Figure 6a. Assuming that Kasha–Vavilov’s rule holds [34], the S–V plots should be somewhat similar in magnitude and shape if FRET is dominant since this phenomenon occurs from the lowest excited state whose quantum yield is independent of the excitation wavelength. Indeed, the S–V plots and magnitudes of *K*_sv_ values are entirely different, and they distinctly depend on the molar extinction coefficient ε of the quencher PA at each excitation wavelength (See Figure 6a). Thus, the smaller response is obtained at λ_exc_ = 285 nm and the larger at λ_exc_ = 371 nm, thus reflecting the growing contribution of primary IFE. The increase of the optical density of the quencher solution as the excitation wavelength approaches the maximum of the absorption spectra of PA (λ_max,abs_ = 355 nm) results in increased absorption of the excitation beam. No second IFE contribution is expected in this case because we observe the decreasing fluorescence intensities at a wavelength (λ_obs_ = 500 nm) where PA absorbance is null. Moreover, S–V plots become increasingly non-linear as the optical density of the solution at the excitation wavelength increases. Likewise, we observed that changing the path-length of the absorption and emission beams inside the cuvette, by placing the film in different sensing geometries, also influenced the film response (Figure S3, Supplementary Materials). Therefore, while the shorter path-length (60°S) into the quencher solution yields a linear S–V plot, longer path-lengths (60°L and 30°) corresponded to higher quenching efficiencies and increasingly non-linear S–V plots, a typical behavior observed when inner filter effects are operative. Therefore, the increase of the optical density either by changing the excitation wavelength or the path-length of the excitation beam results in an upward curvature. In principle, amplified quenching observed in extended conjugated systems [9], or combined quenching that occurs in environments where the fluorophores are confined in a membrane [35], could lead to positive deviations from linearity. In our case, amplified quenching can be ruled out due to the segmented nature of the conjugated system of the polymer **P26** that prevents exciton mobility along the polymer backbone. Although we cannot rigorously exclude that combined quenching occurs after the quencher diffuses into the film, the fact that the above observations and the quenching efficiency order of NACs are correlated with the optical density intensities suggests that IFE plays a dominant role in determining the quenching efficiency order of the polymers at the micromolar concentration range.

**Figure 7:**
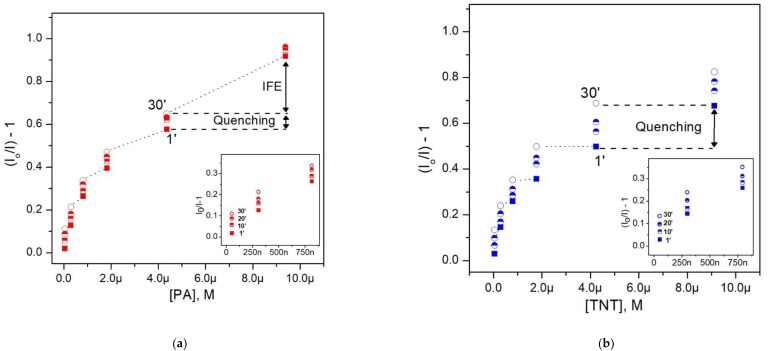
The Stern–Volmer plots for **PN26** films (λ_exc_ = 361 nm, λ_obs_ = 500 nm) with (**a**) picric acid (PA) and (**b**) trinitrotoluene (TNT). Dotted lines are guides to the eye. Dotted lines join the last measurement (after 30 min) at a given concentration and the first (after 1 min) at the next concentration. Insets in the graphs show an expansion of the scale close to the origin.

Next, in order to study the sub-and low micromolar region, thinner films of **P26** (thickness = 35 ± 3 nm) were used to improve quenching efficiencies (Figure S4, Supplementary Materials). Although the effect of thickness of **P26** films on efficiency was not systematically investigated in the present study, we observed the expected improvement in performance by decreasing film thickness, with both quenchers showing remarkable quenching efficiencies in the nanomolar region, as well as similarity in the response towards TNT and PA (Figure 7). Though the non-linear nature of the responses complicates the calculation of the limit of detection (LOD) by linear regression, we observe that 10% of the fluorescence was quenched by 50 nM concentrations of either PA or TNT, so the LOD should be below these concentration values (see insets in Figure 7). Thus, the sensitivities of **P26** are among the best reported in the literature for polymeric solutions or films [25,36,37,38,39].

Besides, fluorescence intensities were measured at 1, 10, 20 and 30 min after the addition of the aliquot of the quencher solution to estimate both the true quenching (ET and/or FRET) and IFE contributions to the total response. Figure 7a shows that the response of the **P26** film to TNT can be exclusively assigned to quenching since the last measurement of fluorescence intensity (after 30 min) at a given concentration and the first (after 1 min) at the next concentration have nearly the same value indicating that there is no sudden signal attenuation due to quencher absorption (i.e., no IFE) after the quencher addition. For each quencher concentration, the film response increases in time due to quenching by diffusion of the NAC into the film. On the other hand, steep growth in the film response occurs immediately after each increment of PA concentration (Figure 7b), which is then followed by slow growth of the response with time due to quenching following diffusion of the NAC into the film. These sudden increments of quenching began to be noticeable at concentrations of 0.3–0.8 µM and already become very important at the low end of the micromolar region. By adding up the quenching increments at each concentration, we estimated that quenching contributes to nearly 50% of the overall response needed to reach *Q*_50%_ at [PA] = 10 µM while for TNT, which is devoid of IFE, *Q*_50%_ is reached at [TNT] = 16 µM (Figure 7). The observed IFE contributions of PA are rooted in the high molar attenuation coefficient of PA in water at λ_exc_ = 371 nm, which is 13,680 M^−1^ cm^−1^ (Figure S5, Supplementary Materials). Therefore, a 5 µM solution would already have an optical density of 0.07, which is significant considering that for analytical studies it is recommended to correct IFE above optical densities of 0.02 [40]. As a result, the sensing response of **P26** towards PA, a quencher that strongly absorbs at the excitation wavelength, has an IFE contribution even at the nanomolar range, while the response towards the non-absorbing TNT depends only on quenching.

## 4. Conclusions

Two new fluorescent segmented conjugated polymers with distyrylnaphthalene chromophores and their model compounds were synthesized. No marked differences in optical properties, morphology or quenching efficiencies were produced by changing the connectivity of naphthylene units. Supported films of both **P14** and **P26** were amorphous, strongly fluorescent with no aggregation of the chromophores in the solid state and highly sensitive towards NACs in water. In particular, PA and TNT showed remarkable quenching efficiencies at the nanomolar level. Quenching experiments using either different nitroaromatic quenchers, excitation wavelengths, excitation beam path-lengths, or time of exposure of the film to the quenching solution point to a dominant role of inner filter effects (IFE) in the quenching efficiency order of the polymer response to PA, TNT, and other NACs in the micromolar concentration range. It was also observed that the sensing response of **P26** towards PA, a quencher that strongly absorbs at the excitation wavelength, has an IFE contribution in addition to quenching even at the nanomolar range, while the response towards the non-absorbing TNT depends only on the quenching occurring after diffusion of the analyte into the film. Finally, we point out here that the adjustment of the optical properties (λ_abs_ and λ_em_) of the polymer by structural design and the judicious selection of experimental conditions (λ_ex_ and λ_obs_) could be used to maximize or minimize IFE as an additional criteria in order to enhance sensitivity or selectivity towards the NAC of interest.

## Supplementary Materials

The following are available online at http://www.mdpi.com/2073-4360/10/12/1366/s1, Experimental 1. General methods and instrumentation, Experimental 2. Synthesis, Figure S1. Optical micrograph (cross polarizers) of P26 between glass substrates al 196 °C, Figure S2. Expansion of the scale close to the origin of Figure 4. Stern–Volmer plots for a PN26 film with nitrotoluenes, Figure S3. Stern–Volmer plots for a PN26 film with PA (λ_exc_ = 371 nm, λ_em_ = 500 nm). The path-lengths of the excitation (blue) and emission (red) beams inside of the cuvette were changed by placing the film in different sensing geometries: 60°L, 30° and 60°S, Figure S4. Fluorescence spectra change (λ_ex_ = 371 nm) of P26 films as a function of (a) added TNT in water; [TNT] = 0.05–189 µM (top to bottom). (b) added PA in water; [PA] = 0.05 –194 µM (top to bottom), Figure S5. Calculation of the molar attenuation coefficient of PA at λ371, Table S1. Thermal and optical properties of model compounds and polymers, Table S2. Data analysis of Stern–Volmer plots and quenching efficiencies, Q_50%_, of the fluorescence responses of P14 and P26 to NACs in water.

## References

[1] Islam M.R., Lu Z., Li X., Sarker A.K., Hu L., Choi P., Li X., Hakobyan N., Serpe M.J. (2013). Responsive polymers for analytical applications: A review. Anal. Chim. Acta.

[2] Juang R.-S., Wen H.-W., Chen M.-T., Yang P.-C. (2016). Enhanced sensing ability of fluorescent chemosensors with triphenylamine-functionalized conjugated polyfluorene. Sens. Actuators B.

[3] Sun X., Wang Y., Lei Y. (2015). Fluorescence based explosive detection: From mechanisms to sensory materials. Chem. Soc. Rev..

[4] Shanmugaraju S., Mukherjee P.S. (2015). π-Electron rich small molecule sensors for the recognition of nitroaromatics. Chem. Commun..

[5] Ding L., Fang Y. (2010). Chemically assembled monolayers of fluorophores as chemical sensing materials. Chem. Soc. Rev..

[6] Squeo B.M., Gregoriou V.G., Avgeropoulos A., Baysec S., Allard S., Scherf U., Chochos C.L. (2017). BODIPY-based polymeric dyes as emerging horizon materials for biological sensing and organic electronic applications. Prog. Polym. Sci..

[7] Ali M.A., Chen S.S.Y., Cavaye H., Smith A.R.G., Burn P.L., Gentle I.R., Meredith P., Shaw E. (2015). Diffusion of nitroaromatic vapours into fluorescent dendrimer films for explosives detection. Sens. Actuators B.

[8] Guan W., Zhou W., Lu J., Lu C. (2015). Luminescent films for chemo-and biosensing. Chem. Soc. Rev..

[9] Rochat S., Swager T.M. (2013). Conjugated amplifying polymers for optical sensing applications. ACS Appl. Mater. Interfaces.

[10] Schwarzenbach R.P., Egli T., Hofstetter T.B., Von Gunten U., Wehrli B. (2010). Global water pollution and human health. Annu. Rev. Environ. Resour..

[11] Rodgers J.D., Bunce N.J. (2001). Treatment methods for the remediation of nitroaromatic explosives. Water Res..

[12] Del Rosso P.G., Romagnolli M.J., Almassio M.F., Barbero C.A., Garay R.O. (2014). Diphenylanthrylene and diphenylfluorene-based segmented conjugated polymer films as fluorescent chemosensors for nitroaromatics in aqueous solution. Sens. Actuators B.

[13] Almassio M.F., Romagnoli M.J., Schvval A.B., Del Rosso P.G., Garay R.O. (2017). Distyrylbenzene-based segmented conjugated polymers: Synthesis, thin film morphology and chemosensing of hydrophobic and hydrophilic nitroaromatics in aqueous media. Polymer.

[14] Mazumdar P., Maity S., Shyamal M., Das D., Sahoo G.P., Misra A. (2016). Proton triggered emission and selective sensing of picric acid by the fluorescent aggregates of 6, 7-dimethyl-2, 3-bis-(2-pyridyl)-quinoxaline. Phys. Chem. Chem. Phys..

[15] Geng T., Zhu Z., Wang X., Xia H., Wang Y., Li D. (2017). Poly{tris[4-(2-Thienyl)phenyl] amine} fluorescent conjugated microporous polymer for selectively sensing picric acid. Sens. Actuators B.

[16] Chowdhury A., Mukherjee P.S. (2015). Electron-rich triphenylamine-based sensors for picric acid detection. J. Org. Chem..

[17] Guo L., Cao D., Yun J., Zeng X. (2017). Highly selective detection of picric acid from multicomponent mixtures of nitro explosives by using COP luminescent probe. Sens. Actuators B.

[18] Tian X., Qi X., Liu X., Zhang Q. (2016). Selective detection of picric acid by a fluorescent ionic liquid chemosensor. Sens. Actuators B.

[19] Duraimurugan K., Balasaravanan R., Siva A. (2016). Electron rich triphenylamine derivatives (D-π-D) for selective sensing of picric acid in aqueous media. Sens. Actuators B.

[20] Wang B., Mu Y., Zhang C., Li J. (2017). Blue photoluminescent carbon nanodots prepared from zeolite as efficient sensors for picric acid detection. Sens. Actuators B.

[21] Sun X., He J., Meng Y., Zhang L., Zhang S., Ma X., Lei Y. (2016). Microwave-assisted ultrafast and facile synthesis of fluorescent carbon nanoparticles from a single precursor: Preparation, characterization and their application for the highly selective detection of explosive picric acid. J. Mater. Chem. A.

[22] Liang H., Yao Z., Ge W., Qiao Y., Zhang L., Cao Z., Wu H.C. (2016). Selective and sensitive detection of picric acid based on a water-soluble fluorescent probe. RSC Adv..

[23] Sahoo J., Waghmode S.B., Subramanian P.S., Albrecht M. (2016). Specific Detection of Picric Acid and Nitrite in Aqueous Medium Using Flexible Eu (III)-Based Luminescent Probe. ChemistrySelect.

[24] Bagheri M., Masoomi M.Y., Morsali A., Schoedel A. (2016). Two Dimensional Host–Guest Metal–Organic Framework Sensor with High Selectivity and Sensitivity to Picric Acid. ACS Appl. Mater. Interfaces.

[25] Tanwar A.S., Hussain S., Malik A.H., Afroz M.A., Iyer P.K. (2016). Inner filter effect based selective detection of nitroexplosive-picric acid in aqueous solution and solid support using conjugated polymer. ACS Sens..

[26] Li W., Wang D., Han D., Sun R., Zhang J., Feng S. (2018). New Polyhedral Oligomeric Silsesquioxanes-Based Fluorescent Ionic Liquids: Synthesis, Self-Assembly and Application in Sensors for Detecting Nitroaromatic Explosives. Polymers.

[27] Ganiga M., Mani N.P., Cyriac J. (2018). Synthesis of Organophilic Carbon Dots, Selective Screening of Trinitrophenol and a Comprehensive Understanding of Luminescence Quenching Mechanism. ChemistrySelect.

[28] Benfaremo N., Sandman D.J., Tripathy S., Kumar J., Yang K., Rubner M.F., Lyons C. (1998). Synthesis and characterization of luminescent polymers of distyrylbenzenes with oligo (ethylene glycol) spacers. Macromolecules.

[29] Neese F., The ORCA program system (2012). Wiley Interdiscip. Rev. Comput. Mol. Sci..

[30] Allouche A.R. (2011). Gabedit—A graphical user interface for computational chemistry softwares. J. Comput. Chem..

[31] Grimme S., Brandenburg J.G., Bannwarth C., Hansen A. (2015). Consistent structures and interactions by density functional theory with small atomic orbital basis sets. J. Chem. Phys..

[32] Bencheikh F., Duché D., Ruiz C.M., Simon J.J., Escoubas L. (2015). Study of Optical Properties and Molecular Aggregation of Conjugated Low Band Gap Copolymers: PTB7 and PTB7-Th. J. Phys. Chem. C.

[33] Lampert Z.E., Reynolds C.L., Papanikolas J.M., Aboelfotoh M.O. (2012). Controlling morphology and chain aggregation in semiconducting conjugated polymers: The role of solvent on optical gain in MEH-PPV. J. Phys. Chem. B.

[34] Lakowicz J.R. (2006). Principles of Fluorescence Spectroscopy.

[35] Schibilla F., Stegemann L., Strassert C.A., Rizzo F., Ravoo B.J. (2016). Fluorescence quenching in β-cyclodextrin vesicles: Membrane confinement and host–guest interactions. Photochem. Photobiol. Sci..

[36] Ghorpade T.K., Palai A.K., Rath S.K., Sharma S.K., Sudarshan K., Pujari P.K., Patri M., Mishra S.P. (2017). Pentiptycene-tbutylpyrene based poly (arylene-ethynylene) s: Highly sensitive and selective TNT sensor in aqueous as well as vapor phase. Sens. Actuators B.

[37] Dong W., Fei T., Scherf U. (2018). Conjugated polymers containing tetraphenylethylene in the backbones and side-chains for highly sensitive TNT detection. RSC Adv..

[38] Han T., Zhang Y., He B., Lam J., Tang B. (2018). Functional Poly (dihalopentadiene) s: Stereoselective Synthesis, Aggregation-Enhanced Emission and Sensitive Detection of Explosives. Polymers.

[39] Mondal S., Jana A., Bera R., Das N. (2017). Design and synthesis of triptycene based fluorescent polymer with pendent triazole: Effect of functionality on host–guest interaction. J. Polym. Sci. Part A Polym. Chem..

[40] Borissevitch I.E. (1999). More about the inner filter effect: Corrections of Stern–Volmer fluorescence quenching constants are necessary at very low optical absorption of the quencher. J. Lumin..

