# Supplementary material for: Fluorescent Amorphous Distyrylnaphthalene-Based Polymers: Synthesis, Characterization and Thin-Film Nanomolar Sensing of Nitroaromatics in Water
Source: Polymers (Basel). 2018 Dec 10;10(12):1366. doi: 10.3390/polym10121366 (PMC6401803; doi:10.3390/polym10121366)
Supplement: Supplementary file 1 [file polymers-10-01366-s001.pdf]

Supplementary Materials

Fluorescent amorphous distyrylnaphthalene-based polymers: Synthesis, characterization and thin-film nanomolar sensing of nitroaromatics in water

Raúl O. Garay*, Ana B. Schvval, Marcela F. Almassio, Pablo G. Del Rosso, Maria J. Romagnoli and Rosana S. Montani

INQUISUR, Departamento de Química, Universidad Nacional del Sur (UNS)-CONICET, Av. Alem 1253, B8000CPB, Bahía Blanca, Argentina.

Experimental 1. General methods and instrumentation

Melting points reported were not corrected. ^{1}H NMR (300 MHz) and ^{13}C NMR (75 MHz) spectra were recorded on a Bruker AVANCE III spectrometer at 25 °C. FT-IR spectra were recorded on a FT-IR Nicolet spectrometer in KBr. Elemental analyses (C, H) were performed on an EXETER CE-440 instrument. Gel permeation chromatography analyses were carried out on THF solutions at room temperature using a Waters model 600 equipped with a Waters 2487 UV detector set at 254 nm, calibration was done using polystyrene standards. Thermal analysis was carried out on a TA Q20 instrument under nitrogen flow at a scan rate of 5 °C/min. The thermal behavior was also observed on an optical polarizing microscope (Leitz, Model Ortolux) equipped with a hot stage (Mettler). UV-vis spectra were obtained from a Cary 60 spectrometer. The absorption measurements were done either on dilute samples (less than 0.01 g/ml) or on thin films cast on quartz plates placed at a 30º angle with respect to the incident beam. Steady-state fluorescence was carried out on dilute samples (less than 0.02 mg/ml) using an SML AMINCO 4800 spectrofluorimeter keeping the optical densities below 0.1 to minimize aggregation and reduce artifacts introduced by self-absorption in fluorescence. Thin film spectra were recorded by front-face (30°) detection.

The films were cast onto carefully leveled quartz or glass substrates (2.54x0.1.1 cm) by spreading over the whole area 0.1 ml of a chloroform solution of the compound. The film was allowed to evaporate slowly in a nitrogen filled chamber and finally was kept under vacuum for 12 hours at room temperature. The thickness of cast films was measured in at least nine different regions using a UV-visible interferometer (Model F20-UV; Filmetrics, Inc.) operated in reflectance mode with a small spot fiber optic, spot size = 0.2 mm. Stock solutions of each NAC were prepared by dissolving the NAC in 3 mL of MeOH and by completing the volume up to 10 mL with water, then further dilutions were made with water. Quenching experiments were performed by inserting the films down to two-thirds of the height of the fluorescence cell. The 1 cm quartz cell was then filled with the solvent (2.4 mL) and spectra were acquired at room temperature by front-face detection at 30° after the addition of microliter aliquots of various hydrophilic and hydrophobic nitroaromatics.

Experimental 2. Synthesis

2.1 Synthesis of bisphosphonates **1a** and **1b**

Bisphosphonates were synthesized by a literature method [25] heating under reflux for 24 h a solution of the aromatic dibromide (24.0 mmol) in triethylphosphite (50.4 mmol) under nitrogen atmosphere.

1,4-Bis(diethoxyphosphinylmethyl)naphthalene, **1a**, was recrystallized in cyclohexane to yield a whitish solid. Yield: 71%; mp: 40-42 °C. ^{1}H NMR ($CDCl_3$) δ: 8.13 (dd, 2H, *J* = 3.2 Hz, *J* = 6.6 Hz), 7.55 (dd, 2H, *J* = 3.2 Hz, *J* = 6.6 Hz), 7.44 (s, 2H), 3.93 (m, 8H), 3.62 (d, 4H, $J_{H\text{-}P}$ = 20.4 Hz), 1.15 (t, 12H, *J* = 7.1 Hz). ^{13}C NMR ($CDCl_3$) δ: 132.4, 127.8, 127.7, 125.8, 125.0, 62.1, 30.9 ($J_{C\text{-}P}$ = 140.3 Hz), 16.2.

2,6-Bis(diethoxyphosphinylmethyl)naphthalene, **1b**, was recrystallized in a mixture of cyclohexane:benzene (5:3) to yield a whitish solid. Yield: 87%; mp: 142-144 °C. ^{1}H NMR ($CDCl_3$) δ: 7.74 (d, 2H, *J* = 8.4 Hz), 7.73 (s, 2H), 7.42 (d, 2H, *J*= 8.4 Hz), 4.02 (m, 8H), 3.29 (d, 4H, $J_{H\text{-}P}$ = 21.4 Hz), 1.23 (t, 12H, *J* = 7.1 Hz). ^{13}C NMR ($CDCl_3$) δ: 132.4, 129.1, 128.3, 128.2, 127.8, 62.2, 34.0 (d, $J_{C\text{-}P}$ = 138.4 Hz), 16.3.

2.2 Synthesis of *E*,*E*-1,4-bis(5-tert-butyl-2-methoxystyryl)naphthalene (**M14**)

NaH 60% (0.223 g, 5.6 mmol) was washed 5 times with dry hexane under Ar atmosphere and suspended in dry DMF (5 mL). Then a solution of **1a** (0.539 g, 1.26 mmol) in dry DMF (6 mL) was added drop-wise under Ar. The solution turned from yellowish to redish color. This mixture was stirred for 3 h and then a solution of **2** (0.51 g, 2.65 mmol) in dry DMF (6 mL) was added under Ar atmosphere. The solution was stirred at room temperature overnight, and quenched by the addition of water (3 mL) and HCl 5% (1.0 mL), extracted with $CHCl_3$ (3x10 mL), dried with Na_2SO_4 and evaporated *in vacuo*. The resulting solid was purified by column chromatography using $CHCl_3$ as eluent. Yield: 320 mg, 52%. mp: 60-65 °C. ^{1}H NMR ($CDCl_3$) δ: 8.28 (dd, 2H, *J* = 6.6 Hz, *J* = 3.2 Hz), 7.93 (d, 2H, *J* = 16.0 Hz), 7.80 (s, 2H), 7.69 (d, 2H, *J* = 2.3 Hz), 7.55 (dd, 2H, *J* = 6.6 Hz, *J* = 3.2 Hz), 7.49 (d, 2H, *J* = 16.0 Hz), 7.30 (dd, 2H, *J* = 8.6 Hz, *J* = 2.29 Hz), 6.89 (d, 2H, *J* = 8.6 Hz), 3.90 (s, 6H), 1.38 (s, 18H). ^{13}C NMR δ: 155.1, 143.4, 135.4, 131.7, 127.2, 126.3, 126.2, 125.7, 125.6, 124.4, 124.1, 123.7, 110.8, 55.7, 34.2, 31.6. FT-IR (KBr, cm^{-1}): 3039, 2955, 2863, 1247, 1031, 966, 811. Anal. Calcd for $C_{36}H_{40}O_2$: C 85.87, H 7.99, O 6.34; found: C, 84.56, H 7.71.

2.3 Synthesis of E,E-2,6-bis(5-tert-butyl-2-methoxystyryl)-naphthalene (**M26**)

The procedure used for the model compound **M14** (see above) was repeated for **M26** with NaH (0.232 g, 5.8 mmol) in DMF (5 mL) and **1b** (0.535 g, 1.25 mmol) in DMF (7 mL). In this case the solution turned from reddish to brownish color. Then, **2** (0.508 g, 2.64 mmol) in DMF (6 mL) was added. Yield: 329 mg, 49%. mp: 204-206 °C. ^{1}H NMR (300 MHz, $CDCl_3$, δ): 7.85 (s; 1H), 7.76-7.78 (2H), 7.65 (d, 2H, *J* = 2.3 Hz), 7.59 (d, 2H, *J* = 16.4 Hz), 7.28 (dd, 2H, *J* = 8.6 Hz, *J* = 2.3 Hz), 7.28 (d, 2H, *J* = 16.4 Hz), 6.86 (d, 2H, *J* = 8.6 Hz), 3.90 (s, 6H), 1.37 (s, 18H). ^{13}C NMR (75 MHz, $CDCl_3$, δ): 155.0, 143.4, 135.6, 133.3, 128.9, 128.2, 126.2, 125.8, 125.6, 124.4, 124.2, 123.6, 110.7, 55.7, 34.2, 31.6. FT-IR (KBr, cm^{-1}): 3009, 2956, 2858, 1275, 1251, 1179, 1023, 971, 811. Anal. Calcd for $C_{36}H_{40}O_2$: C 85.87, H 7.99, O 6.34; found: C, 84.56, H 7.71.

2.4 Synthesis of poly(2,2′′-dimethoxy-*E*,*E*-1,4-distyrylnaphthalene-5,5′′ylene)propylene (**P14**)

To a solution of **1a** (0.500 g, 1.26 mmol) in a 1:1 mixture of THF and DMF (6 mL) 60% sodium hydride oil dispersion (91 mg, 3.78 mmol) was added under nitrogen atmosphere. After cooling the mixture to 0 °C, a solution of **3** (0.394 g, 1.26 mmol) in 1:1 THF-DMF (5 mL) was added drop-wise, then the reaction mixture was allowed to reach room temperature and stirred for 12 h at the same temperature. Afterward, a solution of the end-capping agent diethyl phenylphosphonate (14 mg, 0.063 mmol) in 1:1 THF-DMF (1.2 mL) was added and

the mixture was stirred for an additional 3 h. Benzaldehyde (13 mg, 0.126 mmol) in dry 1:1 THF-DMF (1.2 mL) was then added, the mixture was stirred overnight and quenched with water (20 mL). After neutralization with HCl 35%, a yellow solid was obtained. The polymer was purified by fractional precipitation from $CHCl_3$-methanol. Yield: 294 mg, 60%. ^{1}H NMR ($CDCl_3$) δ: 8.22 (dd, 2H, *J* = 7.7 Hz, *J* = 3.7 Hz), 7.89 (d, 2H, *J* = 16.1 Hz), 7.62 (d, 2H, *J* = 2.4 Hz), 7.76 (s, 2H), 7.48 (dd, 2H, *J* = 7.7 Hz, *J* = 3.7 Hz), 7.46 (d, 2H, *J* = 16.1 Hz), 7.14 (dd, 2H, *J* = 7.9 Hz, *J* = 2.39 Hz), 6.85 (d, 2H, *J* = 7.9 Hz), 3.88 (s, 6H), 1.79 (s, 6H). ^{13}C NMR ($CDCl_3$, 5% DMF-d_7) δ: 155.1, 142.9, 135.1, 131.4, 127.3, 127.0, 126.3, 125.8, 125.6, 125.4, 124.2, 123.4, 110.6, 55.5, 41.8, 31,0.FT-IR (KBr, cm^{-1}): 3049, 2955, 2824, 1250, 1120, 1021, 961, 805. Anal. Calcd. for $C_{31}H_{28}O_2$: C 86.08, H 6.52, O 7.40; found: C 84.35, H 7.13.

2.5 Synthesis of poly(2,2′′-dimethoxy-*E*,*E*-2,6-distyrylnaphthalene-5,5′′ylene)propylene (**P26**)

The procedure used for polymer **P14** was repeated for **P26**. **1b** (0.500 g, 1.26 mmol) in dry THF-DMF 1:1 (12 mL), sodium hydride (0.097 g, 3.78 mmol), **3** (0.394 g, 1.26 mmol) in 6 mL THF-DMF (1:1). Diethyl phenylphosphonate (0.014 mg, 0.063 mmol) in dry THF-DMF 1:1 (1.2 mL), benzaldehyde (0.013 g, 0.126 mmol) in dry THF-DMF 1:1 (1.2 mL). Yield: 277 mg, 55%. ^{1}H NMR (300 MHz, $CDCl_3$, δ): 7.80 (s, 2H), 7.74 (s, 4H), 7.58 (d, 2H, *J* = 2.7 Hz), 7.55 (d, 2H, *J* = 13.5 Hz), 7.23 (d, 2H, *J* = 13.5 Hz), 7.11 (dd, 2H, *J* = 8.4 Hz, *J* = 2.7 Hz), 6.82 (d, 2H, *J* = 8.4 Hz), 3.88 (s, 6H), 1.75 (s, 6H). ^{13}C NMR (75 MHz, $CDCl_3$-5% DMF-d_7, δ): 155.0, 142.9, 135.4, 133.1, 128.9, 128.1, 127.2, 126.0, 125.5, 124.7, 124.2, 124.0, 110.6, 55.5, 41.8, 31.0.FT-IR (KBr, cm^{-1}): 3044, 2959, 2822, 1243, 1029, 965, 808. Anal. Calcd. for $C_{31}H_{28}O_2$: C 86.08, H 6.52, O 7.40; found: C 84.49, H 7.24.

Figures and Tables

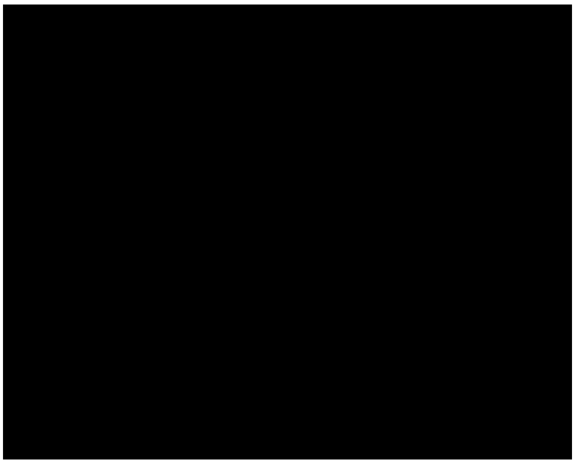

Figure S1. Optical micrograph (cross polarizers) of **P26** between glass substrates at 196 °C.

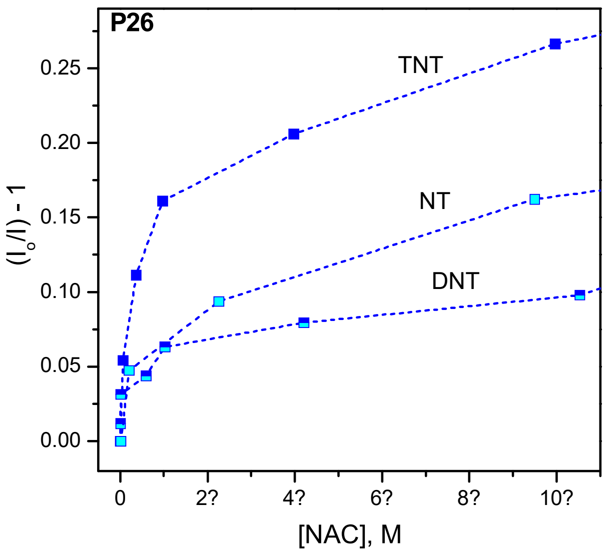

Figure S2. Expansion of the scale close to the origin of the Figure 4. Stern-Volmer plots for a **PN26** film with nitrotoluenes.

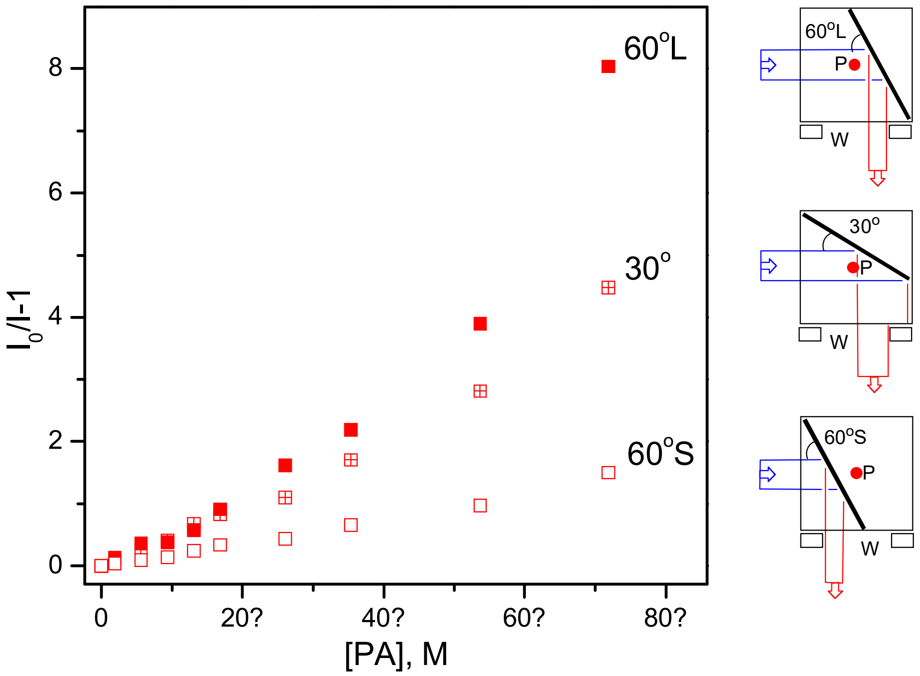

Figure S3. Stern-Volmer plots for a **PN26** film with PA (λ_{exc} = 371 nm, λ_{em} = 500 nm). The path-lengths of the excitation (blue) and emission (red) beams inside of the cuvette were changed by placing the film in different sensing geometries: 60°L, 30° and 60°S. The middle point of the cuvette is marked with the letter P. Fluorescence measurements were done one minute after the quencher addition.

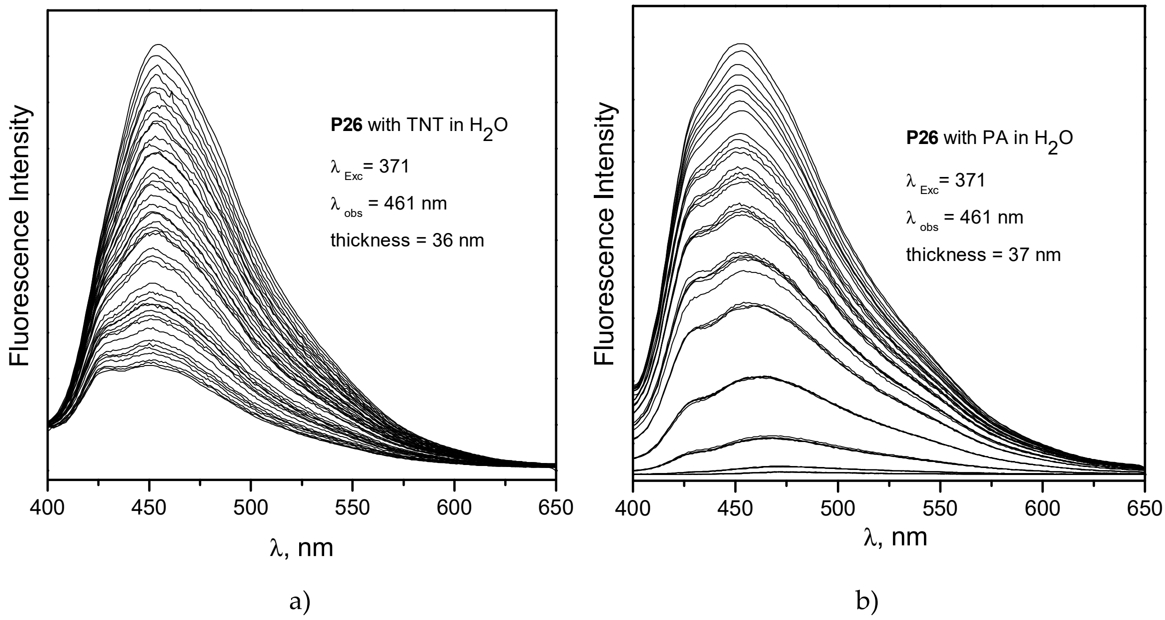

Figure S4. Fluorescence spectra change (λ_{ex} = 371 nm) of **P26** films as a function of a) added TNT in water; [TNT] = 0.05 – 189 μM (top to bottom). b) added PA in water; [PA] = 0.05 – 194 μM (top to bottom).

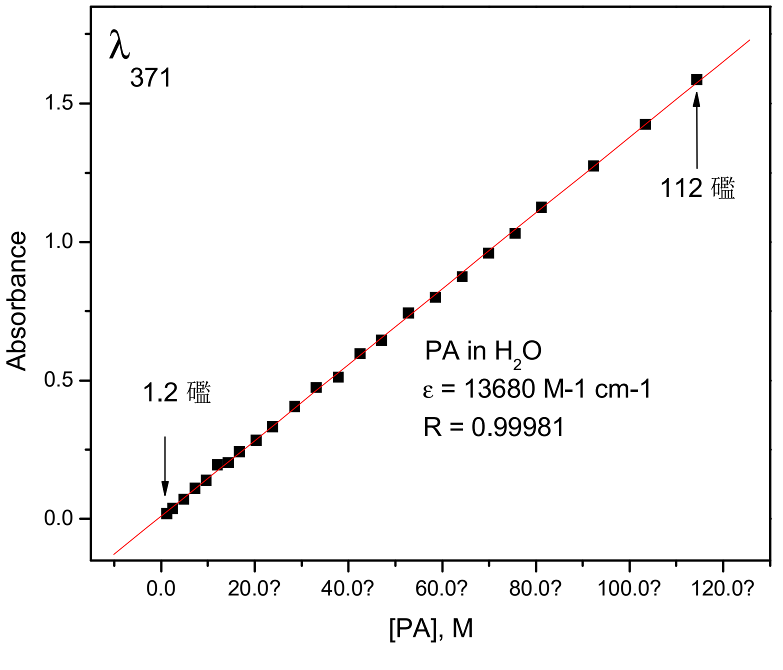

Figure S5. Calculation of the molar attenuation coefficient of PA at λ_{371}. Determination of the lineal range of the PA absorbance in water at the excitation wavelength used to gather fluorescence data from **PN26**.

Table S1. Thermal and optical properties of model compounds and polymers

	DSC[a]	Abs.			Fluo.			
	Tg	media[b]	λ_{max}[c]	fwhm[d]	λ_{max}[e]	fwhm[f]	SS[g]	‹r›[h]
**M14**	30	$CHCl_3$	382	4810	459,	3120	3060	-
		film	383	8540	482	2650	1270	-
**P14**	155	$CHCl_3$	385	7580	465	3200	3780	-
		film	384	7550	472	4410	2930	0.142
**M26**	140	$CHCl_3$	371	4470	434	2500	3500	-
		film	377	10930	467	2650	1870	-
**P26**	170	$CHCl_3$	375	4860	440,	2430	3550	-
		film	378	6630	453	2780	5920	0.119

a. Determined at scan rates of 5 ºC/min, in ºC. Tg = Glass transition temperature from second heating cycle.
b. Measured from dilute $CHCl_3$ solutions and pristine thin films.
c. Absorption maxima measured in dilute $CHCl_3$ solutions and on films, bold data indicate the major peaks.
d. Full width at half-maximum of the absorption bands (in cm^{-1}).
e. Emission maxima measured in dilute $CHCl_3$ solutions and on films. Bold data indicate the major peaks.
f. Full width at half-maximum of the fluorescence bands (in cm^{-1}).
g. Stokes shifts in dilute $CHCl_3$ solutions and on films in cm^{-1}.
h. Average anisotropy measured in thin films in an emission range of 60 nm around the λmax, em.

Tabla S2. Data analysis of Stern-Volmer plots and quenching efficiencies, $Q_{50\%}$, of the fluorescence responses of **P14** and **P26** to NACs in water[a)]

Quencher	K_{sv}[b)]	R[c)]	Linear range[d)]	$Q_{50\%}$[e)]
		**P14**		
NT	1500	0.994	37-63	365
DNT	6780	0.997	43-71	98
TNT	1410	0.993	15-44	422
NF	2460	0.978	42-63	322
DNF	43600	0.997	35-93	23
PA	77750	0.998	0-56	14
		**P26**		
NT	2840	0.999	18-57	290
DNT	3510	0.996	25-64	230
TNT	5070	0.994	34-56	160
NF	12200	0.996	4-65	88
DNF	27100	0.986	9-63	39
PA	22800	0.983	7-55	37

[a)] λ_{exc} = 371 nm, thickness = 108 ± 5 nm. [b)] In M^{-1}. [c)] Correlation coefficient of the linear least squares regression. [d)] Linear range, expressed in % of quenching. [e)] [Q] for (I_o/I) - 1 = 1, μM.
